# A rapid and inexpensive one-tube genomic DNA extraction method from *Agrobacterium tumefaciens*

**DOI:** 10.1007/s13205-013-0132-6

**Published:** 2013-04-28

**Authors:** Suresh P. Kamble, Madhukar M. Fawade

**Affiliations:** 1 Center for Biotechnology, Pravara Institute of Medical Sciences, Loni, Ahmednagar, Maharashtra India; 2 Department of Biochemistry, Dr. Babasaheb Ambedkar Marathwada University, Aurangabad, 411004 Maharashtra India

**Keywords:** Genomic DNA extraction, Restriction digestion, Calcium chloride, Lysozyme

## Abstract

Many methods have been used to isolate genomic DNA, but some of them are time-consuming and costly, especially when extracting a large number of samples. Here we described an easy protocol using two simple solutions for DNA extraction from *A. tumefaciens* cells. Compared with the standard protocol, this protocol allows rapid DNA isolation with comparable yield and purity at negligible cost. Following this protocol, we have demonstrated: (1) gDNA extraction was achieved within 15 min; (2) this method was cost-effective, since it only used calcium chloride and lysozyme; SDS, phenol, chloroform and proteinase K were not necessary; (3) the method gave high yield of gDNA (130 ng/loopful culture) compared with standard protocol that was suitable for restriction analysis; (4) the protocol can be carried out in a single test tube and the cells directly from solid media can be used. Thus, this protocol offers an easy, efficient and economical way to extract genomic DNA from *A. tumefaciens*.

## Introduction

To study the molecular systematics of any organism, high quality DNA is required. The rapid availability of genomic DNA is necessary for cloning genes, selecting recombinant constructs and for taxonomy (Niemi et al. 2001). The cell wall is the main obstacle for quick and easy lysis of *Agrobacterium* cells, and therefore, it must be disrupted for efficient recovery of genomic DNA (gDNA). Conventional methods for gDNA preparation from *Agrobacterium* utilize either enzymatic degradation followed by lysis of cells with detergent or extraction of gDNA with phenol–chloroform (Charles and Nester 1993). When analyzing a large number of samples, these methods are time-consuming and relatively expensive. For quick genotyping, cells can also be lysed by repeated freeze–thaw cycles in a buffer containing Triton X-100 and SDS, followed by extraction of gDNA with chloroform (Harju et al. 2004; Smith and Cantor 1987). Although this method gives good yield, it requires transfer of the sample to a new eppendorf tube after chloroform extraction, which slows down the protocol and makes it inconvenient for simultaneous handling of large number of samples. The above method gives relatively low yield and the results are poorly reproducible. In addition, a large number of cells are required for the protocol.

As calcium chloride is used commonly in *Agrobacterium* transformation protocol to weaken cell walls (McCormack et al. 1998; Mattanovich et al. 1989), we decided to combine it with lysozyme to develop a quick, efficient and robust method for gDNA extraction from *Agrobacterium.*

## Materials and methods

### Culture maintenance and growth conditions

*Agrobacterium tumefaciens* strain C58C1 was grown on LB agar plates (10 g/L tryptone, 5 g/L yeast extract, 10 g/L NaCl, pH 7.2, and 15.0 g/L agar) for 24 h at 28 °C. For gDNA isolation, 48 h culture was used. And for isolation of gDNA from liquid culture, *A. tumefaciens* cells were grown overnight at 28 °C at 200 rpm in LB medium (10 g/L tryptone, 5 g/L yeast extract, 10 g/L NaCl, pH 7.2). 3 ml culture was used.

### DNA isolation

Eight to ten single colonies of *A. tumefaciens* were picked up from LB plate, suspended in 100 μL of 200 mM CaCl_2_ and 1 % lysozyme and incubated at 42 °C for 2–5 min. After incubation, 300 μL of 96 % ethanol was added; the samples were mixed briefly by vortexing; and DNA was collected by centrifugation at 13,200 rpm for 5 min. Precipitated DNA was air dried at room temperature for 10 min and dissolved in 50 μL TE; cell debris was spun down by brief centrifugation at 12,000 rpm for 2 min and supernatant containing purified DNA was directly used for the subsequent experiments or stored at −20 °C.

### Quantification

The purity and yield of gDNA were assessed spectrophotometrically by calculating the *A*_260_/*A*_280_ and *A*_260_/*A*_230_ ratios and *A*_260_ values to determine protein impurities and DNA concentration.

### Restriction analysis

To test whether the gDNA prepared using this method could be digested with restriction enzyme, 1–2 μg of gDNA from *A. tumefaciens* was incubated with 5U EcoRI in a final volume of 20 μL for 2 h at 37 °C and applied to 1 % agarose gel electrophoresis.

## Results and discussion

**Fig. 1:**
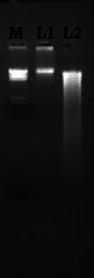
Genomic DNA isolated from *Agrobacterium* by standard and recommended method. M-*Hin*dIII digested Lambda DNA as marker; L1-Genomic DNA isolated by standard method; L2-Genomic DNA isolated by recommended method

In the recommended DNA extraction protocol, *A. tumefaciens* cells were lysed by calcium chloride along with lysozyme without the use of phenol, Triton X-100. Since calcium chloride is used to weaken the cell wall and lysozyme to break up the cell wall (Ledeboer et al. 1976; Chassy 1976; Chassy and Giuffrida 1980), it could directly loose and disrupt the cell wall or nucleus envelop and gDNA was released from the cells. The released gDNA was directly precipitated using 96 % ethanol, omitting phenol chloroform extraction step. This method gave reproducible yields of high quality DNA (Table 1). We also compared our results with the standard method of gDNA extraction protocol (Slusarenko 1990). The obtained genomic DNA by our method and standard method was run in 0.8 % TAE-agarose gel (Fig. 1).

**Table 1:** Yield and quality of DNA obtained from *Agrobacterium* using recommended method

Method	Mean DNA yield (ng/ml)	*A*_260_/*A*_280_	*A*_260/230_
Standard method	150 ± 0.400	1.72	2.185
Recommended method	130 ± 0.325	1.65	1.988

Next, we optimized the protocol to find out the critical components for effective DNA extraction by CaCl_2_–lysozyme lysis method. We tested different concentrations of CaCl_2_ and lysozyme in the lysis solution (data not shown). We also used the different incubation time ranges from 2 to 5 min (data not shown). To summarize, we recommend using 200 mM CaCl_2_ and 1 % lysozyme in the lysis solution and carrying the lysis at 42 °C for 3 min.

**Fig. 2:**
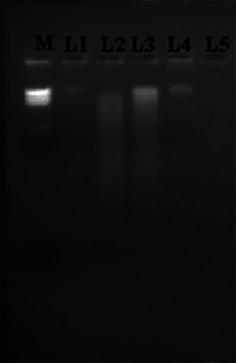
Agarose gel electrophoresis of *EcoR*I restriction digestion reaction of gDNA by standard and recommended method. M-*Hin*dIII digested Lambda DNA as marker; L1-uncut DNA from standard method; L2-digested DNA from standard method; L3-digested DNA from recommended method; L4-Uncut DNA obtained by recommended method; and L5-blank

We used the gDNA prepared by our method for restriction digestion. The restriction digestion pattern of gDNA clearly showed that gDNA obtained could be digested by EcoRI (Fig. 2). The size of most digested gDNA fragments ranged from 23.13 to 0.5 kbp, while the size of control DNA (lane no. 4 in Fig. 2 and lane no. 2 in Fig. 1) corresponded to more than 23 kbp (Fig. 1). Hence DNA was completely digested and there was no evidence of the presence of nucleases in the sample.

We repeated the restriction digestion experiment over a period of 1–2 months and obtained the same banding pattern which indicated the reproducibility of the results and integrity of the gDNA (Ellsworth et al. 1993).

These restriction digestion results show that no restriction process was inhibited by any components in the DNA preparation. This gDNA extraction method has several advantages. First, the numbers of extraction steps were minimized so the gDNA extraction was achieved within 15 min, while other methods needed at least 5–30 min. Second, the method gave high yield of gDNA compared with standard protocol. Third, this method was cost-effective, since it only uses calcium chloride and lysozyme. SDS, phenol, chloroform and proteinase K were not necessary. Fourth, the protocol can be carried out in a single test tube and the cells directly from solid media cab be used.

## Conclusions

We have developed a quick and reliable method for gDNA extraction from *Agrobacterium* that is suitable for restriction digestion. The protocol can be carried out in a single eppendorf tube <15 min and directly from the cells. Finally, it can be used for sequencing, PCR and blotting techniques.

## Abbreviations

gDNA: Genomic DNA
LB: Luria Bertani broth
kbp: Kilo base pairs
CaCl_2_: Calcium chloride
TE: Tris-EDTA
TAE: Tris-acetate EDTA

## References

[CR1] Charles TC, Nester EW (1993). A chromosomally encoded two-component sensory transduction system is required for virulence of *Agrobacterium tumefaciens*. J Bacterio.

[CR2] Chassy BM (1976). A gentle method for the lysis of oral Streptococci. Biochem Biophys Res Commun.

[CR3] Chassy BM, Giuffrida A (1980). Method for the lysis of gram-positive, Asporogenous bacteria with lysozyme. Appl Environ Microbiol.

[CR4] Ellsworth DL, Rittenhouse D, Honeycutt RL (1993). Artificial variation in randomly amplified polymorphic DNA banding patterns. BioTechnique.

[CR5] Harju S, Fedosyuk H, Peterson KR (2004). Rapid isolation of yeast genomic DNA: burst n’ grab. BMC Biotechnol.

[CR6] Ledeboer AM, Krol AJ, Dons JJ, Spier F, Schilperoort RA, Zaenen I, Van Larebeke N, Schell J (1976). On the isolation of TI-plasmid from *Agrobacterium tumefaciens*. Nucleic Acids Res.

[CR7] Mattanovich D, Rüker F, Machado AC, Laimer M, Regner F, Steinkuehler H, Himmler G, Katinger H (1989). Efficient transformation of *Agrobacterium spp.* by electroporation. Nucleic Acids Res.

[CR8] McCormack AC, Elliott MC, Chen DF (1998). A simple method for the production of highly competent cells of *Agrobacterium* for transformation via electroporation. Mol Biotechnol.

[CR9] Niemi RM, Heiskanen I, Wallenius K, Lindstrom K (2001). Extraction and purification of DNA in rhizosphere soil samples for PCR-DGGE analysis of bacterial consortia. J Microbiol Methods.

[CR10] Slusarenko AJ (1990). A rapid mini prep for the isolation of total DNA from *Agrobacterium tumefaciens*. Plant Mol Biol Rep.

[CR11] Smith CL, Cantor CR (1987). Purification, specific fragmentation, and separation of large DNA molecules. Methods Enzymol.

